# Too Good to Be True: Rhesus Monkeys React Negatively to Better-than-Expected Offers

**DOI:** 10.1371/journal.pone.0075768

**Published:** 2013-10-09

**Authors:** Emily J. Knight, Kristen M. Klepac, Jerald D. Kralik

**Affiliations:** Department of Psychological and Brain Sciences, Dartmouth College, Hanover, New Hampshire, United States of America; Institut Pluridisciplinaire Hubert Curien, France

## Abstract

To succeed in a dynamically changing world, animals need to predict their environments. Humans, in fact, exhibit such a strong desire for consistency that one of the most well-established findings in social psychology is the effort people make to maintain consistency among their beliefs, attitudes, and behavior. However, displeasure with unpredictability leads to a potential paradox, because a positive outcome that exceeds one’s expectations often leads to increased subjective value and positive affect, not the opposite. We tested the hypothesis that two evolutionarily-conserved evaluation processes underlie goal-directed behavior: (1) consistency, concerned with prediction errors, and (2) valuation, concerned with outcome utility. Rhesus monkeys (*Macaca mulatta*) viewed a food item and then were offered an identical, better, or worse food, which they could accept or reject. The monkeys ultimately accepted all offers, attesting to the influence of the valuation process. However, they were slower to accept the unexpected offers, and they exhibited aversive reactions, especially to the better-than-expected offers, repeatedly turning their heads and looking away before accepting the food item. Our findings (a) provide evidence for two separable evaluation processes in primates, consistency and value assessment, (b) reveal a direct relationship between consistency assessment and emotional processes, and (c) show that our wariness with events that are much better than expected is shared with other social primates.

## Introduction

To succeed in a challenging and uncertain world, animals need to anticipate change and estimate the likelihood of possible events, both to avoid harm (*e.g.*, a stealthy predator) and to obtain necessary resources that will provide more energy than is expended acquiring them (*e.g.*, catching prey or traveling to distant, ephemeral resources). That is, animals need to make predictions about their environments to determine the appropriate course of action to reach their goals. In fact, there is substantial evidence for the primacy of prediction in human and nonhuman animals, showing, for example, (1) negative emotional reactions to unpredictable events, such as unpredictable shock, with the unpredictability and subsequent uncontrollability being a source of neurosis and depression [1], [2], [3], [4], [5], [6], [7]; (2) neuronal activation in response to novel, unpredictable and surprising events in the amygdala, agranular insula and anterior cingulate cortex independent of positive or negative valence [4], [8], [9], [10]; and (3) that learning is a function of prediction error and surprise [11], [12], [13], [14], [15], [16], [17], [18], [19], [20].

Moreover, the desire to predict accurately appears to be the underlying reason people strive for consistency, either between their beliefs and reality or among their own beliefs, attitudes, and behavior (given that internal inconsistencies suggest inaccuracy). This desire for consistency is one of the most well-established findings in cognitive and social psychology, accounting for multiple phenomena including cognitive dissonance (*i.e.*, one’s reaction to inconsistencies among one’s beliefs, attitudes, and behavior), confirmation biases (*i.e.*, the tendency to overweight evidence that supports one’s current attitudes and beliefs), and the preference for familiarity [21], [22], [23], [24], [25], [26], [27]. Strong evidence for consistency theory was provided by Carlsmith and Aronson [23] who found that after training human participants to expect a bitter or sweet fluid based on a prior cue, then giving them the opposite fluid, the bitter fluid tasted more bitter when expecting the sweet fluid than when expecting the bitter one, and, importantly, the sweet fluid tasted *less* sweet when expecting the bitter one than when expecting the sweet fluid.

Some evidence for the desire for consistency has also been found in a nonhuman primate, the tufted capuchin monkey (*Cebus apella*). In a standard cognitive dissonance test, three equally preferred items are used, where A = B = C, and subjects are forced to choose between A and B. If A is chosen, for example, cognitive dissonance ensues because the actual preference (A = B) does not match choice (A>B). To resolve this dissonance, the subjective value of B is reduced to bring preferences in line with actual choice behavior. In the capuchin study, differently-colored M&M’s® were used [28]. When forced to choose between two equally-preferred colors, the capuchins later appeared to lower the value of the unchosen color by subsequently preferring the third colored M&M over the previously unchosen one. Thus, the capuchins appeared to eliminate the inconsistency between their initial preferences and their choice by devaluing the originally unchosen item. In this general cognitive dissonance test, if all three items were initially of equal value, the test demonstrates that preferences can be directly affected by actual choices. However, in practice, there is a potential problem. If there happened to be subtle differences in preference among the three items, choice could be based simply on subjective value maximization and not the elimination of cognitive dissonance [29], [30].

To address this issue, a subsequent study with capuchins was conducted, this time using differently-colored Skittles® candies [31]. In Condition 1, the ‘choice’ condition, each subject appeared to be given a choice between two options when in actuality the choice was fixed by the experimenter, a perceived ‘choice’ condition. That is, the experimenters hid two items, A_1_ and B_1_, in front of a monkey and the subject was allowed to search for either. Unbeknownst to the subject, however, only one, say A_1_, was available to be found and thus ‘chosen’. In this way, the experimenters could control for the actual choice and thus remove the possibility the capuchins would always choose a color they actually preferred. The monkeys were then given a preference test between the ‘unchosen’ item, B_1_,and a novel one, C_1_. In Condition 2, the ‘no choice’ condition, they were first shown two items, A_2_ and B_2_, but were only offered A_2_. Note that the acceptance of A_2_ in this case did not imply A_2_> B_2_ based on choice because A_2_ was not chosen over B_2_. They were then given the choice between B_2_ and C_2_. Finally, the percentage C_1_ was chosen over B_1_, *i.e.*, C_1_%, was compared to the percentage C_2_ was chosen over B_2_ *i.e.*, C_2_%. The extent to which C_1_%>C_2_% should indicate the influence of cognitive dissonance, which was 60% to 49% in the study. Thus, this finding again suggests that the capuchin monkeys may have eliminated the inconsistency between their initial lack of preference and choice behavior by devaluing the unchosen food item and thus preferring the novel item to bring their preferences in line with their choices. Hence, there is some evidence for consistency assessment in nonhuman animals with respect to preference and choice behavior. However, it remains unclear whether consistency is sought in other domains, most notably, in predictions versus actual events.

Of course, animal learning as well as human judgment and decision-making research have also established the importance of prediction, showing that the valuation of an outcome (*i.e.*, the assessment of its subjective value or utility) depends on what was expected. Consequently, for example, a positive outcome is subjectively less positive if something better was expected: e.g. receiving $10 when $100 was expected [32], [33], [34], [35]. Outcome valuation, then, produces an overall subjective value that is based on both the objective value received and the subjective sense of whether the outcome was a gain or loss compared to what was expected [36], [37]. Even so, the valuation process assesses outcomes on a continuous scale, meaning that in general higher value is better; and thus, it is useful to conceptualize the goal of the valuation process as the maximization of subjective value (or utility).

There is, then, an apparent contradiction between the desire for consistency, on the one hand, and the desire to maximize subjective value, on the other. To see this, consider, for example, encountering a better-than-expected outcome, such as someone unexpectedly offering to pay your restaurant dinner check. Based on consistency theory, your reaction should be negative, given that the outcome was not anticipated. In contrast, based on maximization of subjective value, your reaction should be positive, given that you unexpectedly saved money.

The contradiction is reconciled if we posit that both evaluation systems underlie goal-directed behavior: a consistency assessment reflected in an immediate affective response based on how well a given event or outcome was predicted [21], [22], [23], [24], [25], [26], [27], and a value assessment in the subsequent affective response and action of approaching (accepting) or avoiding (rejecting) an outcome (offer) [13], [15], [32], [33], [34], [35], [38], [39], [40]. To date, however, evidence for two separable processes and how they may interact remains unclear. In humans, Shepperd and McNulty [41] tested whether, after hearing unexpectedly good or bad news about an exam grade or medical test, responses were best described by consistency or valuation (*e.g.*, utility maximization) theories, and concluded that their evidence supported the latter. They did not, however, test whether both processes might be working in concert, with the consistency process responding first, revealing an initial negative reaction to an unexpected outcome (whether positive or negative), followed by the valuation process, producing a subsequent positive response. Moreover, they used contexts in which valuation may dominate a consistency assessment response, given that exam grades and medical tests scale such that better results are much clearly better than poorer ones [42].

In nonhuman animals, Tinklepaugh [43] found that macaque monkeys reacted negatively when they received a lesser-valued food item (e.g. piece of lettuce) when a higher one was expected (e.g. piece of banana); however, he did not observe a clear response in the reverse condition, in which the higher-valued item was found when the lesser-valued one was expected, stating that this latter condition needs to be further studied, and thus leaving a distinction between consistency and valuation preferences inconclusive.

In the current study, we tested the hypothesis that two evolutionarily-conserved evaluation processes underlie goal-directed behavior: one based on consistency and the other on valuation. We reasoned that if these two processes were separable, the same event, such as a better-than-expected offer, would lead to two opposing reactions: an initial negative, aversive reaction reflecting the prediction error detected by the consistency process, and a subsequent positive reaction reflecting the positive outcome expected by the valuation process, leading to the acceptance of the better-than-expected offer. We tested two rhesus macaques (*Macaca mulatta*) in two experiments that both included expected offers. In addition, the first experiment included a worse-than-expected offer and the second included a better-than-expected one. In all cases, the monkeys accepted the offers. However, both monkeys exhibited initial negative reactions to the unexpected offers, and to our surprise, both monkeys exhibited the strongest negative reaction to the better-than-expected offer.

## Methods

### Ethics Statement

Animal care and use complied with all current laws, regulations, policies, and guidelines of the United States, the United States Department of Agriculture (USDA), the Public Health Service (PHS), and all procedures were approved by the Institutional Animal Care and Use Committee (IACUC) of Dartmouth College.

### Subjects

Two naïve, seven-year old rhesus macaques (*Macaca mulatta*), Hamlet and Puck, were available for the study. They were housed in 32×27×68 (width × depth × height) inch cages (Allentown Inc., Allentown, NJ) in a homeroom with automatically regulated temperature, ventilation, humidity, and lighting (14∶10 hour light:dark cycle, with lights on at 0600 hours). The monkeys were intermittently housed in pairs and individually: at times when they engaged in fighting, which is normal periodic behavior in young rhesus macaque males of similar size and temperament [44], the two monkeys were separated and individually-housed for their safety.

The Center for Comparative Medicine and Research (CCMR) at Dartmouth maintains a full-time animal care and veterinary staff that monitors the monkeys’ daily health and well-being. The monkeys were maintained at approximately 95% of their *ad libitum* weights to ensure sufficient motivation and good health, and their diet consisted of primate chow (no. 5038, PMI Feeds Inc., St Louis, Missouri, U.S.A.), supplemented with fresh fruit and vegetables, as well as various treats that included peanuts, cereal, and dried fruits (*e.g.*, raisins, banana).

To allow for continued social stimulation, the subjects had direct visual contact with the other monkeys in the colony, the animal care staff, and experimenters. When pair-housed, they had direct physical contact with each other, and also when individually-housed, through a mesh grading divider between their cages. In addition, environmental enrichment included two or more enrichment items in their home cages at all times, daily playing of radio or videos in the room (the latter via a monitor mounted in view of all individuals), and regular access to a larger enrichment cage (68×38×72 inch) in an adjacent room.

We subsequently conducted an affective decision-making study with these monkeys as well as with free-ranging rhesus monkeys at the Caribbean Primate Research Center on Cayo Santiago in Puerto Rico [45]. We obtained the same general findings in the laboratory and field, suggesting that the laboratory conditions are not significantly biasing experimental results, and that the subjects in the current study are representative of rhesus monkeys in general [46].

### Materials and Food Items

To obtain precise response times, the study used a button panel with two convex Plexiglas-covered buttons (approximately 16 cm apart measured from the centers). Both buttons had lights mounted inside of them, the left was red, the right blue. The buttons were clear colored when not lit, red and blue, respectively, when lit. For the preference test (described below), the buttons were positioned just to the left and right of center. For the remainder of the study, only the red button was used, and the button panel was positioned with the red button in front of the monkey. Two food items were used: a 45-mg cereal pellet (Bio-Serv, Frenchtown, NJ, USA) and a miniature marshmallow.

### General Procedure

The monkeys were brought to the testing room in the laboratory individually in custom-made chairs. The chairs were designed for maximal comfort and safety, in which the monkey’s collar slid into a slot that placed the monkey in their preferred natural sitting position, on a perch off the floor. The chair loosely restrained the left arm of the monkey while allowing free movement of the right arm. The monkeys were progressively acclimated to the chairs by (a) initially having them sit near a chair and eat treats (*e.g.*, raisins, peanuts, fresh fruit and vegetables) placed on it; and then (b) feeding the monkeys treats when they were first seated in the chair. After acclimation, the monkeys readily entered the chair; and once seated, they exhibited no signs of stress and displayed natural behaviors such as facial expressions and vocalizations, *e.g.*, food grunts. For the current study, the chairs were used to maintain precise experimental control over food item presentation and obtain precise response time measurements, with the monkey’s right hand starting at the same position and the presented food item and button at a fixed position relative to the monkey on every trial. Similar chairs are routinely used in monkey neuroeconomic studies that have successfully replicated multiple behavioral phenomena studied with other paradigms, both in the laboratory and field [47], [48].

Each monkey was tested separately, and sat across a standard black laboratory table from the experimenter, with the button panel placed in front of the monkey. To minimize the possible cuing of the subjects by the experimenter, we enacted a number of procedures, including (a) the experimenter wearing a white lab coat, goggles, medical mask and gloves to mask visual cues; (b) playing white noise to mask auditory cues; (c) the experimenter following a well-practiced, timed, and stereotyped movement to present the food items; and (d) using an automated system to record response times. We also note that it would be unlikely that the complex reactions exhibited by the monkeys would be due to experimenter cuing. Finally, for all training and testing procedures (described below), the intertrial interval was approximately ten seconds.

### Preference Test Training

Prior to the preference test, both subjects were trained with the button panel. The experimenter turned the light on one of the buttons (pseudo-randomly determined), placed a pellet in her hand, closed her hand, placed it behind one of the buttons (from the monkey’s perspective), and then opened it. The monkey was required to press the button in front of the pellet to receive it. Once the pellet was taken, the experimenter turned off the button light and the intertrial interval began. This procedure was repeated until the monkeys learned to press the button upon receiving the offered pellet, *i.e.*, when the experimenter opened her hand revealing the pellet. Although these training sessions were not recorded, both monkeys required approximately 10 sessions to learn the task, with up to 50 trials per session.

### Preference Test

For the 50-trial preference test, on every trial, the experimenter turned on both button lights, placed one food item in each hand (left/right position of the items pseudo-randomly determined), closed her hands, placed them behind each button (from the monkey’s perspective), and then opened them. The monkey chose the preferred food item by pressing the corresponding button in front of it. The monkey was given the chosen food item; the experimenter then turned off both button lights and the intertrial interval began.

### General Task Description

The general task for the monkeys was to observe a single food item for three seconds, which would potentially establish an expectation [37], observe the food item then being removed, observe the red light inside the button turning on to signal that the next presented food item would be an offer, and then once the second item was presented, press the button if the offer is accepted (or refrain from pressing it if the offer is rejected). We denote this trial sequence as ‘displayed food item’ followed by ‘offered food item’: *i.e.*, ‘displayed food item→offered food item’.

### General Task Training Procedure

Prior to the experimental manipulations, the monkeys were trained on the task paradigm using pellets only; and thus, with a trial sequence ‘pellet→pellet’. The experimenter presented the first pellet by placing her closed left hand just behind the button and opening it in one quick motion. The pellet was presented for the monkey to observe for three seconds to potentially establish the expectation and then the hand was closed and pulled back. She then turned on the red light in the button to cue the monkey of an impending offer, and presented the second pellet identically as the first. The second pellet was given to the monkey upon pressing the button. This procedure was repeated until the monkeys learned to respond only after the red light was lit and the second pellet was presented as the offer. Although these training sessions were not recorded, both monkeys required approximately 10–15 sessions to learn the task, with approximately 50 trials per session.

### General Task Testing Procedure

After training was complete, the monkeys received five 50-trial familiarization sessions, conducted on separate days, to acclimate them further to the test paradigm, as well as to provide standardized, baseline experience prior to conducting the two experiments. For the first 25 trials, we again displayed a single pellet for three seconds to establish an expectation, removed it, and then offered an identical pellet (*i.e.*, ‘pellet→pellet’). A monkey accepted the second pellet by pressing the button, and we recorded his reactions electronically via response times and video. If the monkey did not press the button within 30 seconds, the response was considered a rejection of the offered food item. Thus, it is important to note that the monkeys could reject the offer by simply not responding. For the second 25 trials, the experimenter first displayed a marshmallow for three seconds, turned on the red light to signify the impending offer, and then offered a marshmallow: ‘marshmallow→marshmallow’. These trial types were tested in two blocks of 25 trials with ‘pellet→pellet’ first to minimize the potential influence of marshmallows on the motivation to receive pellets (see Table 1 for the trial block structure).

**Table 1:** Block sequence for the Familiarization condition (five consecutive sessions) and Experiments 1 and 2 (one session each), and within the condition and experiments, the trial block offer types and number of trials.

Block	Familiarization Condition	Numberof Trials	Experiment 1	Numberof Trials	Experiment 2	Numberof Trials
1	Pellet → Pellet	25	Pellet → Pellet	25	Pellet → Pellet	25
2	Marshmallow → Marshmallow	25	Marshmallow → Marshmallow	25	Pellet → Pellet	25
			interleaved with		interleaved with	
			Marshmallow → Pellet	25	Pellet → Marshmallow	25
3					Marshmallow → Marshmallow	25

A daily session for each monkey consisted of either the two blocks of the Familiarization Condition, the two blocks of Experiment 1, or the three blocks of Experiment 2.

An inherent difficulty in studying expectancy is that subjects quickly adjust to the violations with experience: that is, the unexpected becomes expected. Therefore, to minimize this effect while also obtaining enough trials for analysis, after the five familiarization sessions, we tested the two critical manipulations (worse-than-expected and better-than-expected offers) in two experiments, each conducted in single sessions on separate days. Experiment 1 consisted of one 75-trial session. As shown in Table 1, the session consisted of the following: (a) one block of 25 ‘pellet→pellet’ trials, followed by (b) a block of 50 trials, in which 25 ‘marshmallow→marshmallow’ trials were randomly interleaved with 25 ‘marshmallow→pellet’ trials, *i.e.*, the unexpectedly worse offer, in which a pellet was offered after first seeing the marshmallow. Experiment 2 consisted of one 100-trial session. Also listed in Table 1, the session consisted of the following: (a) one block of 25 ‘pellet→pellet’ trials, followed by (b) a block of 50 trials, with 25 ‘pellet→pellet’ trials randomly interleaved with 25 ‘pellet→marshmallow’ trials, *i.e.*, the unexpectedly better offer, in which a marshmallow was offered after first seeing the pellet, and (c) a final block of 25 ‘marshmallow→marshmallow’ trials. To minimize the potential influence of marshmallows on motivation to receive pellets, we conducted the 25 ‘pellet→pellet’ trials first in all sessions, and the 25 ‘marshmallow→marshmallow’ trials last in Experiment 2. Thus, the first block of 25 ‘pellet→pellet’ trials in Experiment 2 were used in the analysis.

With respect to data analysis, it has been argued that the best procedure to manage dependent variable outliers, such as response times, in distributions that will be compared is *trimming*, which treats all conditions consistently by removing the same number of highest and lowest values from all response time samples; we adopted the procedure here and removed the highest five and lowest five response times for every offer type [49], [50], [51]. We then used two-tailed student’s *t*-tests for comparisons between offer types. Note that after using the trimming method, the degrees of freedom for the offer type comparisons were 15+15–2 = 28. Means are reported with the standard error of the mean (SEM).

## Results

**Figure 1:**
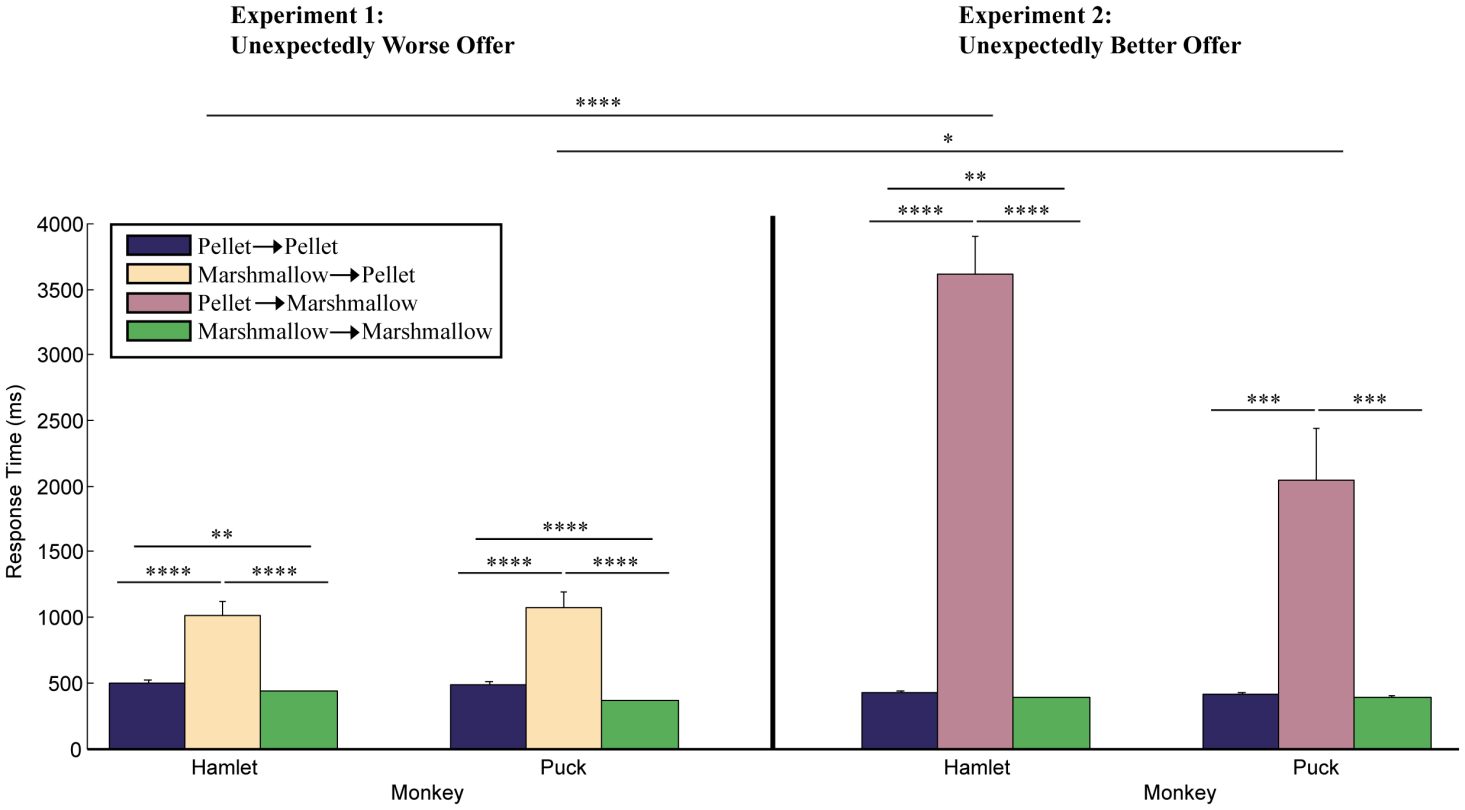
The average response time (ms) from the presentation to the acceptance of the offered food item for each offer type for both monkeys. Results shown for two-tailed student’s *t* test, ‘*’ = *p*<0.05, ‘**’ = *p*<0.01, ‘***’ = *p*<0.001, ‘****’ = *p*<0.0001.

To verify preference for the marshmallow over the pellet, we first conducted the 50-trial preference test, and both monkeys chose the marshmallow over the pellet every trial (two-tail binomial test, *n = *50, *p*<0.0001). Then, after training on the general task, and then five familiarization sessions in which the monkeys received a total of 125 ‘pellet→pellet’ and 125 ‘marshmallow→marshmallow’ trials (*i.e.*, viewing a pellet followed by a pellet offer, and viewing a marshmallow followed by a marshmallow offer), both experiments were conducted in back-to-back sessions on separate days. For Experiment 1, the monkeys received three offer types: ‘pellet→pellet’, ‘marshmallow→marshmallow’, and an unexpectedly worse offer: ‘marshmallow→pellet’ (*i.e.*, viewing a marshmallow followed by a pellet offer) (see Table 1). As seen in the left panel of Figure 1, as expected, both monkeys were slower to accept the unexpectedly worse offer compared to the expected ones (see Table 2 for statistical results). On average, both monkeys responded over twice as long to the unexpected offer compared to the expected ones. In addition, reflecting the monkeys’ preference for the marshmallow, the response times were faster for the ‘marshmallow→marshmallow’ trials than for the ‘pellet→pellet’ ones (Figure 1, Table 2).

**Table 2:** Results obtained for all offer types in Experiments 1 and 2, including response times (mean ± SEM), and student’s *t* test and *p* values for each offer type comparison.

	Experiment 1				Experiment 2			
Comparison	Hamlet		Puck		Hamlet		Puck	
	ResponseTimes (ms)	*t*(28)value *p*<	ResponseTimes (ms)	*t*(28)value *p*<	ResponseTimes (ms)	*t*(28)value *p*<	ResponseTimes (ms)	*t*(28)value *p*<
Marshmallow → Pellet	1016±110	4.64	1077±121	4.8				
Pellet → Pellet	501±17	0.0001	486±24	0.0001				
Marshmallow → Pellet	1016±110	5.29	1077±121	5.89				
Marshmallow → Marshmallow	433±9	0.0001	364±7	0.0001				
Marshmallow → Marshmallow	433±9	3.55	364±7	4.89	386±7	3.08	394±8	1.29
Pellet → Pellet	501±17	0.01	486±24	0.0001	426±10	0.01	412±12	ns
Pellet → Marshmallow					3611±296	10.75	2049±395	4.14
Pellet → Pellet					426±10	0.0001	412±12	0.001
Pellet → Marshmallow					3611±296	10.89	2049±395	4.19
Marshmallow → Marshmallow					386±7	0.0001	394±8	0.001

In Experiment 2, the monkeys again received three offer types: ‘pellet→pellet’, ‘marshmallow→marshmallow’, and an unexpectedly better offer: ‘pellet→marshmallow’ (*i.e.*, viewing a pellet followed by a marshmallow offer) (Table 1). As seen in the right panel of Figure 1, rather than responding faster to the unexpectedly better offer, both monkeys were slower to accept it (see Table 2 for statistical results). On average, Hamlet responded over eight times slower compared to the expected offers, and Puck responded approximately five times slower. Again, reflecting preference for the marshmallow, both monkeys’ response times were faster for the ‘marshmallow→marshmallow’ trials than for the ‘pellet→pellet’ ones, although the difference was significant for Hamlet only (Figure 1, Table 2).

Comparing the responses to the two unexpected offer types, ‘marshmallow→pellet’ and ‘pellet→marshmallow’ from Experiments 1 and 2, respectively, the delay to accept the better-than-expected offer was significantly longer than for the worse-than-expected one, being over three and a half times longer for Hamlet and almost twice as long for Puck (see Figure 1 and Table 2). If the longer delay to accept the better-than-expected offer reflected surprise, excitation, or the need to process the large, positive reward change, we would expect the monkeys to look longer at the unexpected food item, as shown with looking-time paradigms, which are predicated on the fact that humans, including infants and children, as well as nonhuman animals tend to look longer at unexpected, surprising events [52], [53], [54], [55]. In contrast, our monkeys did not look longer at the better-than-expected offers. In fact, they did the opposite, conspicuously averting their eyes and turning away from the unexpectedly better food item (the marshmallow). To obtain the clearest and most conservative measure of this response, we video coded simultaneous head and eye aversions: that is, both (a) the head was turned, and (b) the monkey was not looking at the food item. Two people scored the videos on all conducted trials, one experimentally blind, with a lowest correlation between them of Pearson’s *r*(148) = 0.99, *p*<0.0001. Then, we used the trials in the response-time analyses (*i.e.*, after trimming, described in Methods) to obtain the percentage of time on every trial that the monkeys were averting their head and eyes before responding to the offer. We took the total aversion time on the trial (*i.e.*, amount of time simultaneously turned and looking away) and divided it by the total response time for the trial (between offer and button press) and multiplied by 100. Finally, to obtain the overall percentage for each condition, we took the average percentage of all trials used in the analyses. First, there were no aversions in either experiment for any of the expected offers: *i.e.*, ‘pellet→pellet’ or ‘marshmallow→marshmallow’. Second, both monkeys exhibited aversions to both unexpected offer types: for ‘marshmallow→pellet’ in Experiment 1, Hamlet 9±5% and Puck 3±3% of the time; for ‘pellet→marshmallow’ in Experiment 2, Hamlet 81±2% and Puck 23±8% of the time. For Experiment 1, Hamlet’s aversion rate to the unexpected offer (‘marshmallow→pellet’) was significantly higher compared to the expected ones (*t*(28) = 2.08, *p*<0.05); however, Puck’s was not. For Experiment 2, both monkeys’ aversion rates to the unexpected offer (‘pellet→marshmallow’) were significantly higher compared to the expected ones (Hamlet: *t*(28) = 41.49, *p*<0.0001; Puck: *t*(28) = 2.97, *p*<0.01). Third, both monkeys exhibited higher aversion rates to the better-than-expected food item compared to the worse-than-expected one (Hamlet: *t*(28) = 14.56, *p*<0.0001; Puck: *t*(28) = 2.46, *p*<0.05), with Hamlet’s rate being nine times higher with the better-than-expected food item and Puck’s over seven and a half times higher.

Finally, by definition, the unexpected events should become less surprising with increased exposure, and we found some evidence for this within the session. In Experiment 1, both monkeys’ response times to accept the unexpected offer (‘marshmallow→pellet’) decreased across the session (Linear regression, Hamlet: *R*(14) = 0.88, *p*<0.0001; Puck: *R*(14) = 0.68, *p*<0.01). In addition, in Experiment 2, Hamlet’s response times to accept the unexpected offer (‘pellet→marshmallow’) decreased across the session (Linear regression, *R*(14) = 0.70, *p*<0.01). The only other offer type showing a change across trials was a decrease in response times for Hamlet in the Experiment 1 ‘pellet→pellet’ trials (Linear regression, *R*(14) = 0.58, *p*<0.05). The only change across trials in the video coded aversion rates occurred for Hamlet in the Experiment 1 unexpected offer (‘marshmallow→pellet’) (Linear regression, *R*(14) = 0.73, *p*<0.01). Thus, the simultaneous head and eye aversions of both monkeys for the better-than-expected offer (‘pellet→marshmallow’) in Experiment 2 were generally sustained across the session.

## Discussion

In Experiment 1, as expected, both monkeys were slower to accept the unexpectedly worse offer (‘marshmallow→pellet’) compared to the expected ones. This finding is consistent with evidence that outcome evaluation by humans and nonhuman animals is affected by how the problem scenario is framed [34], [36], [37], [56]. Thus, the first presented food item, such as the marshmallow, appeared to become a reference to the monkeys, which then was used to evaluate the subsequently offered item, such as the pellet. However, from the results of Experiment 1, it remains unclear whether the increased response time reflected a single evaluation process underlying goal-directed behavior or whether there was more than one, in particular, consistency and value assessment. Put differently, it is at this point unclear whether the increased response time was due to the devaluation of the second food item or surprise and potential cognitive dissonance.

In Experiment 2′s key offer type, we first displayed a pellet to the monkeys but then offered a marshmallow. Given that in Experiment 1 they were faster to accept a marshmallow when offered one in the ‘marshmallow→marshmallow’ trials compared to the other offer types, one might have expected a similar result or even a faster response to the better-than-expected offer. Assuming that response time reflects expected value (and there is no floor effect with the monkeys already reaching as quickly as they can in the ‘marshmallow→marshmallow’ trials), a faster response time would be predicted by standard valuation theories (*e.g.*, utility maximization); and in any case, standard valuation theories would not predict a slower response time. Additional factors that could have led to a faster response to the better-than-expected offer include heightened arousal and an increased influence of lower-level prepotent mechanisms causing an immediate reach to the desired food item [57], [58], [59], [60], [61], [62], [63], [64]. However, the opposite occurred in that both monkeys were much slower to accept the marshmallow after first seeing a pellet as compared to the expected offers (*i.e.*, compared to ‘pellet→pellet’ and ‘marshmallow→marshmallow’).

Not only did both monkeys respond more slowly to both the better- and worse-than-expected offers than to the expected ones, they responded even more slowly to the better-than-expected offer than to the worse-than-expected one. One possible reason for the longer response times for the ‘pellet→marshmallow’ offer type (Experiment 2) versus the ‘marshmallow→pellet’ one (Experiment 1) could be context effects, regarding how their experience might have affected expectations differently for the two unexpected offer types. One potential context effect is whether the monkeys were faster to accept the pellet offer in the ‘marshmallow→pellet’ trials compared to the marshmallow offer in the ‘pellet→marshmallow’ trials because they had more experience responding to the pellet (especially in initial training and the first blocks of Experiments 1 and 2). However, even with this extra experience, both monkeys were generally faster to respond to the marshmallow in the ‘marshmallow→marshmallow’ trials; therefore, experience with accepting particular offers did not appear to influence their behavior substantially.

A second potential context effect regards the food item displayed prior to the offered item. More experience with ‘pellet→ pellet’ trials could have led to the displayed pellet becoming a stronger predictor of the subsequent pellet offer (*i.e.*, a stronger discriminative stimulus) compared to the strength of the marshmallow predicting an offered marshmallow. If so, a pellet followed by a marshmallow could have been more surprising, leading to a longer response time. At the same time, we note that it was also possible that prior experience could have had the opposite effect on ‘pellet→marshmallow’ trials, with the monkeys learning prior to Experiment 2 that different combinations of presented and offered items were possible and thus less surprising (having experienced ‘pellet→ pellet’, ‘marshmallow→marshmallow’ and ‘marshmallow→pellet’). Nonetheless, context effects could have contributed to longer response times with the ‘pellet→marshmallow’ offer type; and it will be important in the future to characterize how experience and other contexts effects contribute to such expectation development. As we discuss below, it is also possible that monkeys generally have less experience with better-than-expected outcomes compared to worse-than-expected ones.

However, the main objective of the current study was to determine if any context effects could be demonstrated in rhesus monkeys by setting up potential expectations in both worse-than-expected and better-than-expected directions. The results show that this objective was achieved. In both experiments, the response times of the unexpected offers for both monkeys were significantly different from the expected ones. Although the longer response time to the worse-than-expected offer of a pellet following a marshmallow was anticipated, the underlying reasons for the longer response time to the better-than-expected offer of a marshmallow following a pellet are less clear.

If the longer delay to accept the better-than-expected offer reflected surprise, excitation, or the need to process the large, positive reward change, the monkeys should have looked longer at the unexpected food item, as shown with numerous studies that used the ‘looking-time’ paradigm, in which subjects look longer at unexpected, surprising events [52], [53], [54], [55]. Not only was the marshmallow following the displayed pellet potentially surprising, it might also have produced a positively-valenced affective reaction, which should have focused attention further on the unexpected ‘jackpot’ or ‘prize’. In contrast, our monkeys conspicuously averted their eyes and turned away from the unexpectedly better food item (the marshmallow).

What could have caused the increase in the aversion rate (*i.e.*, percentage of time simultaneously turning their heads and looking away from the marshmallow)? Three possible reasons for turning away from the better-than-expected food item are (1) disinterest, (2) the loss of experimental control of their behavior in a novel situation, or (3) active avoidance of the food item. The first, disinterest, is unlikely given (a) that the monkeys were maintained at 95% *ad libitum* weight to assure sufficient motivation; (b) the generally faster response times with the more highly-preferred food item throughout the experiment (*i.e.*, the ‘marshmallow→marshmallow’ trials); and (c) that neither monkey looked away from the offered food items on the ‘expected’ trials. Thus, the monkeys exhibited interest in the food items and were not simply responding independent of them, for example, based on habit [40], [65]. The second possibility, the loss of experimental control, could occur if the posed problem was sufficiently novel from what they had learned. The novel offer of the marshmallow following the pellet might have left them simply confused, which might have resulted in looking around, being uncertain of what to do. We find this possibility unlikely given that they had extensive training with the task structure, with the red light and second food item signifying an offer. Although it is possible that they could not generalize from the ‘pellet→ pellet’, ‘marshmallow→marshmallow’, and ‘marshmallow→pellet’ trials, it seems less likely given that they readily generalized from the ‘pellet→ pellet’ training to the ‘marshmallow→marshmallow’ trials (first familiarization session response times on the ‘marshmallow→marshmallow’ trials: Hamlet, 624±40 ms; Puck, 383±5 ms, which were both over five times faster than for the better-than-expected ‘pellet→marshmallow’ trials). In addition, if the monkeys did not know what to do, they should not have eventually accepted the offers by pressing the button within a few seconds and should not have subsequently reached toward the experimenter’s hand to obtain the marshmallow, which they both did, appearing to reflect an understanding of the general task structure. Finally, even with a loss of experimental control of their behavior, it seems unlikely that they would look away from the marshmallow, experimenter, and experimental apparatus, given that, in general, there were blank walls to their left and right (and behind them).

We are therefore left with the possibility that the monkeys were actively avoiding the unexpected food items, and in particular, the marshmallow. First, head and eye aversions are a telltale aversive response in rhesus monkeys [44], [66], [67]. Second, the behavior we observed is comparable to that found in affective neuroscience with ‘fear’ tests, in which, for example, monkeys must reach across a transparent box to obtain a desired food item at the back of the top of the box. Inside the transparent box is a fake spider or snake that the monkeys must reach over. Indicators of the emotional fear response to the spider or snake are eye aversions and head turning, which we adopted here [66], [67]. Thus, it appears that both monkeys in our study exhibited a negative reaction to the unexpected food items, and in particular, to the better-than-expected marshmallow. This finding was especially strong with Hamlet when confronted with the better-than-expected marshmallow, in which he turned away from it for long periods of time (over 80% of the time prior to accepting it). Although there is evidence in nonhuman animals for positive and negative contrast effects [68], [69], [70], [71], [72], frustration or disappointment [43], [73], [74], [75], [76], and surprise [8], [11], [16], [18], [19], [76], to our knowledge, this is the first report of nonhuman animals displaying overt negative affect in response to a better-than-expected event.

Although both monkeys reacted negatively to the unexpected outcomes, they, nonetheless, eventually accepted the offers by pressing the button, rather than rejecting them by not pressing the button. One possible explanation for their eventual acceptance of the unexpected offers is if the monkeys pressed the button independent of the offer, for example, as a means to proceed to the next trial or as an automatic habitual response. However, given that the food items were taken and eaten immediately makes this possibility unlikely. In addition, the faster response times to the more preferred marshmallow (in the ‘marshmallow→marshmallow’ trials) than the less-preferred pellets (in the ‘pellet→pellet’ trials), even though they had more experience with ‘pellet→pellet’ trials, also suggest that their behavior was goal-directed, based on the offer value. We take this dichotomy of reactions–initially reacting negatively by looking away and taking longer to respond, but nonetheless eventually accepting the offer and consuming it–as evidence for two processes underlying goal-directed behavior: one based on consistency assessment and the other based on value assessment. Moreover, our results suggest that the consistency process may occur prior to the valuation process, such that individuals compare their expectation to the actual event, and then first react negatively to error (and adjust their expectations), reflecting consistency assessment, and then react positively (if better than expected), reflecting valuation of the expected outcome. Evidence for these two underlying evaluation processes, one based on consistency, the other on value, may not be surprising, given the large literatures supporting each one (as discussed in the introduction) [1], [3], [4], [5], [12], [13], [15], [17], [18], [21], [22], [23], [24], [25], [26], [27], [28], [31], [43]. However, to our knowledge, this is also the first demonstration of the interrelationship of both processes, at least in a nonhuman animal.

Our finding of an aversive reaction to unexpected events also provides evidence for a direct relationship between consistency and emotional processes in the brain, which presumably motivate animals, including humans, to actively minimize the cognitive dissonance of expectancy violations, either via avoiding these contingencies or learning to anticipate them in the future [25], [38], [39], [43], [77], [78], [79], [80], [81], [82]. Moreover, evidence for a separable consistency process and its relationship to emotion provides support for the contention that there are monitoring processes in the brain, for example, to alert higher-level systems to override lower-level ones [4], [8], [9], [10], [83], [84], [85], [86], [87]. For instance, if our monkeys had developed a basic routine to some degree to perform the task, an unanticipated change in the routine could have led a consistency-based monitoring system to generate an alarm signal, as reflected in the negatively-valenced emotional response. This signal could activate other processes, such as higher-level ones, to assess the problem. For example, self-regulation could be applied until it is determined that the valuation system can proceed [88]. In any case, a consideration of consistency and value assessment as separable processes should help clarify complex reactions such as *surprise*, given that the valence of the reaction (*i.e.*, whether positive or negative) to expectancy violation should depend on the timing and relative strengths of the underlying processes [4], [8], [23], [32], [89], [90].

Although we found evidence for the existence of both evaluation processes underlying rhesus monkey goal-directed behavior in both of our subjects, further studies will need to test more individuals to determine the extent to which rhesus macaques in general exhibit responses that reflect a desire for both consistency and positive subjective value. Our study has shown that at least some individual monkeys do, and thus these processes coexist in at least some rhesus macaques. Although we obtained consistent results with both monkeys, one might expect to find individual differences in the relative weighting of the influence of these processes, with some individuals preferring consistency, routine, and exploitation of what is known, and others preferring novelty and exploration to find potentially richer payoffs. For macaque monkeys, this consistency versus novelty seeking distinction may correlate with factors like dominance rank, age, and gender, and may be significantly heritable, given that these general dispositions have a significant genetic component in humans [91], [92].

Future work will also need to clarify the specific conditions under which these effects are elicited. Indeed, different contexts will likely influence the relative weighting of the two processes, as is likely the case with people. Nonetheless, our study has revealed that there are conditions under which both consistency assessment and subjective valuation are manifest in the goal-directed behavior of rhesus macaques.

The negative response of the monkeys to the unexpected events, especially the better-than-expected one, appears to reflect their inexperience with such situations and the resulting discomfort when expectations are violated [1], [3], [4], [5], [12], [13], [15], [17], [18], [21], [22], [23], [24], [25], [26], [27], [28], [31], [43]. Interestingly, in instances in which the outcome is highly unexpected, skepticism may in fact be warranted. Better-than-expected events in particular may be a rarer occurrence, especially in a social environment in which self-regulation may be the norm for most individuals to protect against conflict, severe punishment, and deception. The sentiment that something is *too good to be true* may reflect this inherent wariness or skepticism with large prediction discrepancies in the positive direction [23], [93], [94]. Although this phenomenon is firmly ensconced in popular culture, little is known about the conditions under which an expectancy violation may lead to concerns and wariness about the actual outcome. Here, we have found evidence that the concern people have with events that are much better-than-expected is shared with at least one other social primate. Furthermore, our results suggest that a ‘too good to be true’ effect is not a devaluation of the potential outcome, but rather, a reflection of the primacy of the consistency process whereby one compares the current offer to what is expected. Valuation is then halted while a further evaluation of the situation is made.

Some nonhuman primates have been shown to turn down offers when another individual receives a better one [95], [96], or when offered something better than a social partner [96]. By the same token, some primate species have also demonstrated altruistic acts in which they help others [97], [98], even at some cost to themselves [99], [100], [101]. These instances, together with our findings, demonstrate socio-cognitive processes that interact with the general tendency to maximize value, attesting to the complex contingencies of living in a social world [102], [103]. In fact, the separable process of prediction-based consistency detection and management may be a precursor to more sophisticated higher-order cognitive abilities, including self-regulation, model-based planning and mental simulation, deception detection, and social contract management [20], [21], [24], [26], [27], [34], [102], [103], [104], [105], [106].

The finding that both of our rhesus monkeys exhibited a preference for consistency between what is expected and what is experienced may also have clinical relevance, especially when preference for constancy, routine, and ritual becomes extreme, as observed in obsessive compulsive disorder [107] and the insistence on sameness in autism spectrum disorder [108], [109], [110]. An understanding of the evolutionarily-conserved cognitive and affective mechanisms underlying expectancy violations could account for some of these symptoms. It is indeed likely that there was strong selection pressure on social primates to minimize unpredictability in a complex, dynamic, and uncertain world, and to remain vigilant with events that are worse than expected or others that may be too good to be true.
